# Alexander (W.) on a New Method of Treating Hitherto Incurable Cases of Incontinence of Urine in Women

**Published:** 1888-08

**Authors:** 


Alexander (W.) on a New Method of Treating hitherto Incurable Cases of Incontinence of Urine in Women.—The first case he operated upon was a woman belonging to the theatrical profession, who often had to retain her urine for unduly prolonged periods of time. This ultimately determined a paralysis of the sphincter of the urethra. To remedy the distressing effects of this condition of things various methods of treatment were employed, but in vain, and ultimately he dissected out the urethra, and led it into the rectum, hoping to utilize the rectal sphincter for the retention of urine. At the third attempt he succeeded, and the patient was much relieved. He also read the notes of two other cases in which he had operated for vesico-vaginal fistulæ by closing the vulva and carrying the urine into the rectum.—*Med. Press, May 2, 1888.*

---

INSTEAD of sponges Billroth uses gauze prepared as follows: The absorbent gauze is cut into pieces eight inches square, and of these seven are folded and sewed at their edges. These are boiled twice in one day, one hour each time, in a sublimate solution of 110 degrees. They are then placed in jars and carefully covered. Gauze thus prepared is thoroughly antiseptic. Every attempt with such gauze to cultivate micro-organisms has failed.—*Bull. Med.*

